# Development of a standardized measure to assess food quality: a proof of concept

**DOI:** 10.1186/s12937-016-0215-4

**Published:** 2016-11-09

**Authors:** L. H. Jomaa, N. C. Hwalla, J. M. Zidek

**Affiliations:** 1 Lamis Jomaa, Faculty of Agricultural and Food Sciences, American University of Beirut, Beirut, Lebanon; 2 Nahla Hwalla, Faculty of Agricultural and Food Sciences, American University of Beirut, Beirut, Lebanon; 3 Jeremy Zidek, Futura Food, 113 Creekside Drive, State College, PA 16801 USA

**Keywords:** Novel measure, Food quality, Nutrient density, Nutrient profiling, qCaln

## Abstract

**Background:**

Food-based dietary guidelines are promoted to improve diet quality. In applying dietary recommendations, such as the MyPlate, the number of servings in a food group is the unit of measure used to make food selections. However, within each food group, different foods can vary greatly in their nutritional quality despite often having similar energy (caloric) values. This study aimed to develop a novel unit of measure that accounts for both the quantity of energy and the quality of nutrients, as defined by caloric and micronutrient density, respectively, in foods and to demonstrate its usability in identifying high quality foods within a food group.

**Methods:**

A standardized unit of measure reflecting the quality of kilocalories for nutrition (qCaln) was developed through a mathematical function dependent on the energy content (kilocalories per 100 g) and micronutrient density of foods items within a food group. Nutrition composition of 1806 food items was extracted from the USDA nutrient database. For each food item analyzed, qCaln ratios were calculated to compare qCaln to its caloric content. Finally, a case example was developed comparing two plates adapted from the MyPlate.

**Results:**

Examples of food items with highest and lowest qCaln ratios were displayed for five food groups: vegetables, fruits/fruit juices, milk/dairy products, meats/meat alternatives, and breads/cereals. Additionally, the applicability of the qCaln was presented through comparing two plates, adopted from the USDA MyPlate, to show differences in food quality.

**Conclusions:**

The newly developed qCaln measure can be used to rank foods in terms of their nutrient density while accounting for their energy content. The proposed metric can provide consumers, public health professionals, researchers, and policy makers with an easy-to-understand measure of food quality and a practical tool to assess diet quality among individuals and population groups.

**Electronic supplementary material:**

The online version of this article (doi:10.1186/s12937-016-0215-4) contains supplementary material, which is available to authorized users.

## Background

Identifying best methods to measure food and diet quality is an ongoing scientific and policy-level challenge facing public health professionals, researchers, and decision-makers. Over the years, several approaches and methods have been proposed to assess food and diet quality, including diet quality indices and various nutrient profiling methods.

Diet quality indices were developed to measure conformance of individuals and population groups to international and national dietary guidelines and recommendations, such as the U.S. dietary guidelines (MyPlate) [1]. The Healthy Eating Index (HEI) 2005 and the alternative HEI (AHEI 2010) are examples of diet quality indices that have been extensively used by public health professionals and researchers in a variety of settings and age groups. The HEI 2005 and the more accurate and up-to-date AHEI 2010 are based on the Dietary Guidelines for Americans whereby researchers assess the frequency of consuming food items and the nutrient intake within and among food groups, respectively, compared to these dietary guidelines [2]. Both indices were successful in finding associations between diet and health outcomes, such as obesity and chronic diseases including diabetes, cardiovascular diseases, and cancer [3]. However, limitations exist with these diet quality indices. A major shortcoming is the difficulty of determining which foods within a food group score better than others. When making food selections, it is also not immediately obvious to a consumer that a food will score high or low on the HEI. Furthermore, consumers may not be able to discern which foods within a food group are of high nutritional quality or nutrient density, and which foods are of lower quality, especially for foods that lack nutrition facts labels like fresh fruits and vegetables.

The nutrient profiling method, defined as the science of ranking foods based on nutrient composition [4, 5], represents an alternative approach proposed by researchers to improve assessment of food and diet quality. Numerous methods have been proposed and used by researchers, regulatory agencies, and food industry to develop, test, and validate nutrient profile models [4], however, these approaches still have their limitations. Some nutrient profiling approaches were developed with the aim of increasing healthy micronutrient consumption. Others focused on less desirable nutrients that need to be limited, such as saturated fat, added sugars, and sodium, or a combination of both beneficial and less desirable nutrients that need to be limited [5]. Although various nutrient profiling models have been validated against established indicators of diet quality and can aid in the value-based selection of quality foods [6, 7], a singular based score of quality that can account both for the macro- and micro-nutrient content of foods has yet to be achieved.

Challenges are faced when interpreting currently used diet quality indices and nutrient profiling measures, particularly in non-research settings, when consumers are requested to meet food-based dietary guidelines (FBDGs) and nutrition recommendations. For example, in applying dietary recommendations, such as the US MyPlate [8], the number of servings in a food group is the unit of measure used to make food selections. However, within each food group, different foods can vary greatly in their nutritional quality despite often having similar energy (caloric) values. The complexity of diet quality indices and nutrient profiling approaches hinder consumers’ ability to make healthier dietary choices. This is particularly challenging in the midst of a complex food environment that has a wide array of food products with contradictory health and nutrition claims and, at best, confusing food-labeling systems (front-of-package labels and nutrition facts labels). These environmental conditions coupled with difficult-to-understand diet quality measures add to the confusion of average consumers and may lead to poor dietary choices [9, 10].

In order to address this problem, the present study aimed to create a novel unit of measure that accounts for both the quantity of energy (kilocalories per 100 g) and the quality of nutrients, as defined by micronutrient density, in foods and to demonstrate the usability of this novel measure to facilitate the assessment of food quality. Specific objectives of the study included 1) developing a standardized unit of measure reflecting the quality of kilocalories for nutrition (qCaln) that can account for the caloric and nutrient density per food item, 2) comparing food items per food groups based on the newly developed qCaln measure, and 3) testing the applicability of qCaln measure through demonstrating different food plates with various qCaln ratio scores.

Findings from this study can provide a new measure that allows for merging multiple nutritional parameters into one simple, standardized unit. In addition, this newly proposed measure could provide researchers and public health professionals with a relatively easier tool to assess the diet quality of individuals and population groups in comparison to other nutrient profiling tools and diet quality indices.

## Methods

### Development of qCaln measure

Generally, food energy has been measured in kilocalories (kcal, or Cal), which accounts for the energy in the macronutrients (carbohydrates, fats, and proteins) included in the sum total of ingredients in any food item. The quality of the food, however, is more often than not, determined by the amount and distribution of key micronutrients in food. Energy, macronutrient and micronutrient content of all food items within the Nutritionist Pro software (version 5.1.0, 2014, SR 24, First Data Bank, Nutritionist Pro, Axxya Systems, San Bruno, CA) were included in the computations. The food composition database in this software was based on the United States Department of Agriculture (USDA) National Nutrient Database for Standard Reference, release 27 [11]. In addition, a small number of local foods and beverages commonly consumed among Lebanese and Middle Eastern populations were considered in the calculations and were denoted as (AUB) in the figures, where applicable. Local foods and beverages were compared to culturally-specific food composition tables [12].

The qCaln is a function of the caloric content (per 100 g) from macronutrients and the amount and distribution of micronutrients (per 100 g) in a particular food such that:

1 $$ qCaln = Cal+Cal\ast \left\{{\displaystyle {\sum}_{i=1}^n}{w}_i\ast {S}_i\right\} $$

where “Cal” is the amount of kilocalories per 100 g in the food ingredient, w_i_ is the weighting for each micronutrient I, “S_i_” is the micronutrient score for each micronutrient “I”, and n is the amount of micronutrients included in the calculation. The value w_i_ is the relative weighting for importance to each of the micronutrients.

Equation 1 allows for as many micronutrients as necessary to describe the nutrient density, and the authors recognize that some populations may require different types and distributions of micronutrients. For the purpose of this paper, a total of 10 micronutrients were selected for inclusion in the qCaln formula. The 10 micronutrients that were considered for this paper and their units are: vitamin A (RE), vitamin D (μg), vitamin C (μg), calcium (mg), iron (mg), zinc (mg), dietary fiber (g), saturated fat (g), total sugar (g), and sodium (mg). Total sugar was used instead of added sugar given that data on the latter is currently less readily available, and previous research has shown that using total sugar as a nutrient to limit is a reasonable option [6]. The selection of these nutrients were based on a number of factors: 1) the high prevalence of micronutrient deficiencies at a global level and their serious adverse health effects due, in part, to inadequate dietary intake of certain nutrients, including vitamin A, iron and zinc [13–17]; 2) the strong associations observed between diet-related chronic diseases and the high consumption of saturated fat, sugars, and sodium [18–21]; (3) recommended nutrients or nutrients that are restricted with upper limits according to international food agencies such as the Food and Drug Administration (FDA) in the US [22, 23] and the French Food Standard Agency (AFSSA) [24, 25]; and 4) comparable choice of nutrients reported in previous nutrient profiling indices such as the naturally nutrient rich (NNR) score [26] nutritional quality index (NQI) [27] and ratio of recommended to restricted food components (RRR) [28]. Equal weights for all micronutrients were considered in this study when calculating the qCaln measure. The equal weightings among micronutrients was based on the same assumption as that of the dietary reference intakes (DRIs) developed for healthy people, whereby the same relative importance is assumed among micronutrients [29]. Although some population groups may have additional physiological needs, such as during infancy or pregnancy, and individuals residing in developing nations may be at higher risk of certain micronutrient deficiencies, including iron and zinc deficiencies, the qCaln was developed to address the food quality regardless of the health and physiological status of consumers.

Thus, each micronutrient was assigned an equal weighting *w*_*i*_ of 0.10 as the weighting scores indicate that each micronutrient is equally important in a diet. For the purposes of this study, weightings for the selected micronutrients were considered identical, and weightings for micronutrients that were not included in the calculation were assumed to be “0”. Saturated fat, total sugar, and sodium were negatively weighted in the qCaln calculation, indicating that these micronutrients need to be limited to prevent cardio-metabolic risk factors, including elevated blood sugar, cholesterol, or blood pressure levels [30–33].

Equation 1 can then be expanded such that,

2 $$ \mathrm{qCaln} = \mathrm{C}\mathrm{a}\mathrm{l} + \mathrm{C}\mathrm{a}\mathrm{l} \ast \left\{{w}_i \ast \left({\mathrm{S}}_{\mathrm{VA}} + {\mathrm{S}}_{\mathrm{VD}} + {\mathrm{S}}_{\mathrm{VC}} + {\mathrm{S}}_{\mathrm{Ca}} + {\mathrm{S}}_{\mathrm{Fe}} + {\mathrm{S}}_{\mathrm{Zn}} + {\mathrm{S}}_{\mathrm{DF}}\ \hbox{-}\ {\mathrm{S}}_{\mathrm{satfat}}\hbox{--}\ {\mathrm{S}}_{\mathrm{S}\mathrm{ugar}}\hbox{-}\ {\mathrm{S}}_{\mathrm{Na}}\right)\right\}\ . $$

For each food item, and for each micronutrient “i”, a micronutrient score “S_i_” was calculated. The calculation is based on the amount of the selected micronutrient in the food item relative to other food items in the same food group to ensure proper comparison. The distribution of each selected micronutrient among all foods within each food group was tested for normality and were found to be close to a normal distribution. Given a normal distribution, the value of S_i_ will depend on the standard score (otherwise known as z-score or z-value) of the micronutrient. The equation for the standard score of a micronutrient amount relative to the rest of the food group is given by:

3 $$ z = \frac{x - \mu }{\sigma } $$

where ‘x’ is the amount of a selected micronutrient (for example, “mg per serving size”), μ is the sample mean of the same micronutrient with all food items in that food group, and σ is the sample standard deviation of that micronutrient within the same foods included in the food group. Once the z-score was calculated, the critical value, or percentile score of the value z by integrating for the probability of our test value z being less than Z as Z → ∞, was calculated. This value gives the probability that any statistic is less than the calculated z-score in Eq. 3 and is given by integrating the probability density function,

4 $$ P\left(a\ \le x\ \le b\right) = {\displaystyle \underset{a}{\overset{b}{\int }}f\left(x\ \right)dx} $$

where

5 $$ f(x) = \frac{1}{\sqrt{2\pi {\sigma}^2}}\ {e}^{\frac{-{\left(x-\mu \right)}^2}{2{\sigma}^2}}. $$

Equation 4 represents the area under a continuous curve f (x), and Eq. 5 represents the density function. Equation 5 cannot be integrated in terms of functions that can be expressed as exponentials, polynomials, trigonometric, logarithmic, and rational functions. Therefore, the probability P_i_ of a micronutrient i was estimated based on z-score tables such that (0 ≤ P_i_ ≤ 1). Then, for each micronutrient i,

6 $$ {S}_i=2{P}_{{}_i}-1. $$

Once S_i_ was calculated for each micronutrient i, Eq. 2 was used to calculate the qCaln value for each food item taking into consideration all the selected micronutrients in the sample of food items within that food group. Thus, it follows that foods with critical z scores above the 50^th^ percentile (0.5 < P_i_ ≤ 1.0) will have a greater qCaln value than the listed value of Cal indicating higher quality food, and foods with lower than the 50^th^ percentile (0.0 ≤ P_i_ < 0.5) have qCaln values less than the listed Cal, indicating less quality. Also, it follows from Eqs. 2 and 6 that the minimum qCaln value would approach 0.0 and the maximum value would approach a value twice the amount of Cal (qCaln_max_ = 2 * Cal).

### Calculation of qCaln ratio

After calculating S_i_ for each micronutrient, a qCaln value per 100 g was calculated for 1806 different food items from five different food groups. These 5 main food groups were: 1.) breads and cereals, 2.) dairy products, 3.) fruits and juices, 4.) vegetables, 5.) and meat and meat alternatives. Using the weightings from Eq. 1 and the data from the USDA National Nutrient Database [11], the qCaln ratio, was calculated to compare qCaln to Cal for each food item analyzed. A complete example showing qCaln results for each food within the vegetables food group is shown in an Additional file 1 [see “qCaln-Vegetables.csv”]. Ratios of qCaln that are greater than 1.0 can be interpreted as higher quality food per calorie, whereas qCaln ratios that are less than 1.0 represent lower micronutrient content per food item compared to its caloric content.

7 $$ qCaln\  Ratio=\frac{qCaln}{Cal}. $$

### Comparison of food plates

To show the applicability of the qCaln ratio, two separate plates were compared. Each plate was based on FBDG recommendations from USDA MyPlate [8] and consisted of at least one serving of food from fruits and fruit juices, vegetables, meat and meat alternatives, milk and dairy products, and breads and cereals. Figure 6 shows the two plates selected for comparison. Table 1 lists the contents for each plate compared. Each food item listed was assumed to be one portion size of food. The qCaln ratio was then calculated for each food item and averaged for the entire plate of food items.

**Table 1:** Contents of each USDA MyPlate example for comparison

Food group	Plate 1	Plate 2
Fruits and fruit juices	Apple, cherry, orange, pineapple	Grapes, kiwi, banana
Vegetables	Carrot, green pepper, tomato	Tomato, onion
Breads and cereals	Oat bran muffin, white toasted bread, and wheat (white wheat) bread	White pita bread, spaghetti pasta
Meat and meat alternatives	Egg, pork sausage, bacon	Meatless meatballs (meat alternative), hot dog
Milk and dairy products	Whole milk	Fruit-flavored yogurt

## Results

### Comparison of qCaln of food items within same food groups

**Fig. 1:**
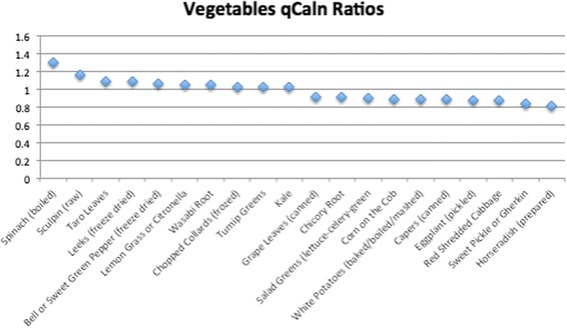
Spectrum of qCaln ratios for selected vegetables

**Fig. 2:**
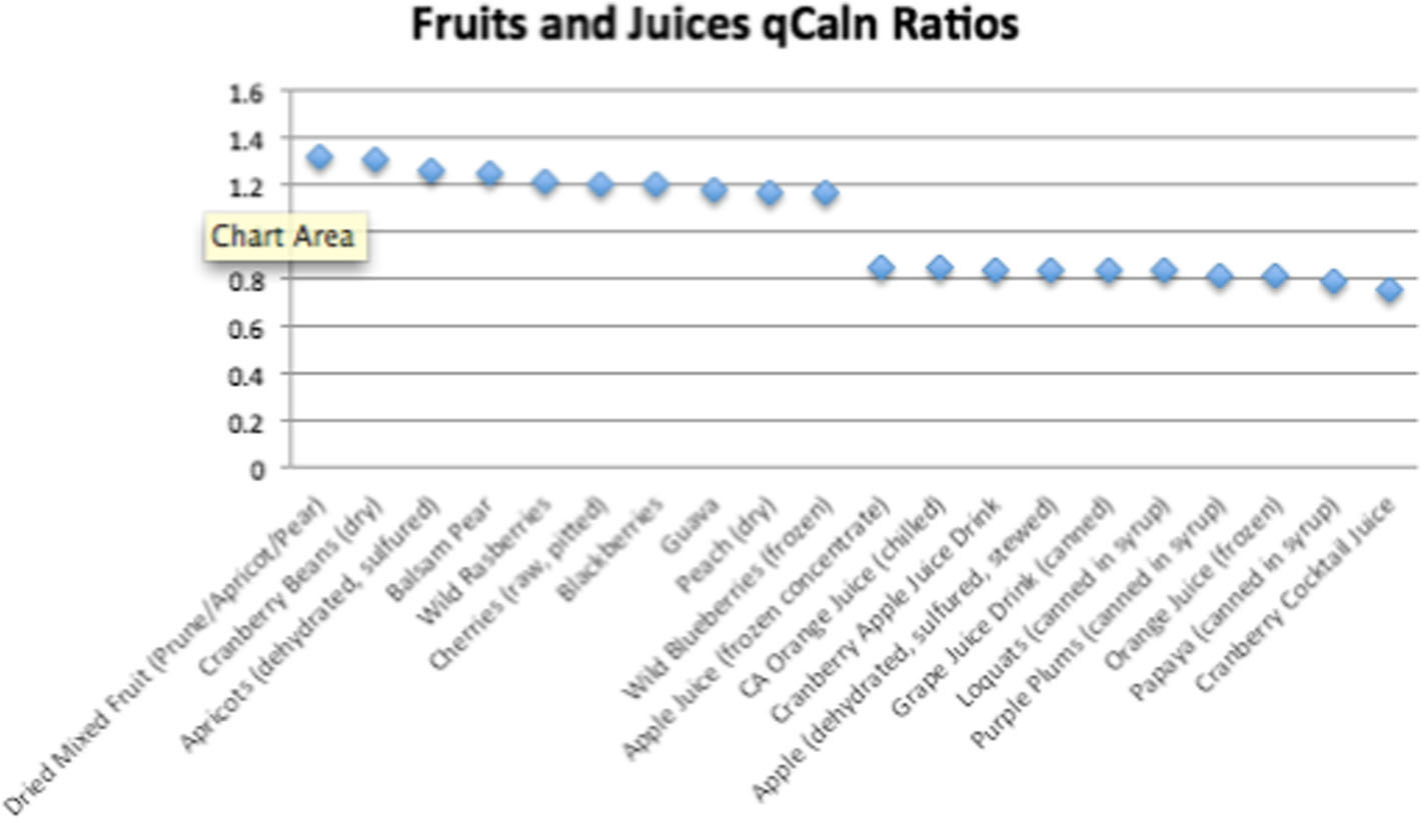
Spectrum of qCaln ratios for selected fruits and fruit juices

**Fig. 3:**
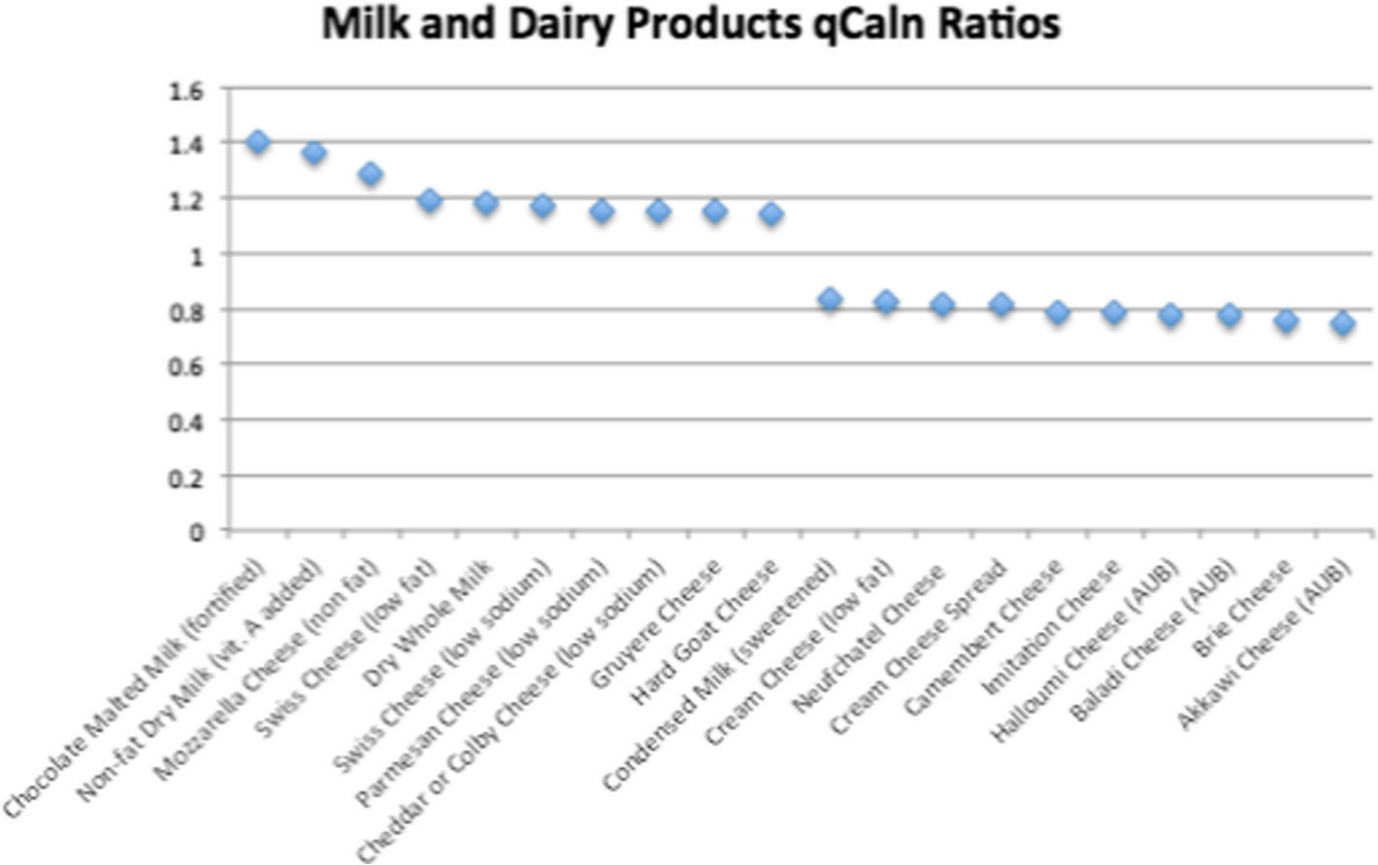
Spectrum of qCaln ratios for foods in the milk and dairy group

**Fig. 4:**
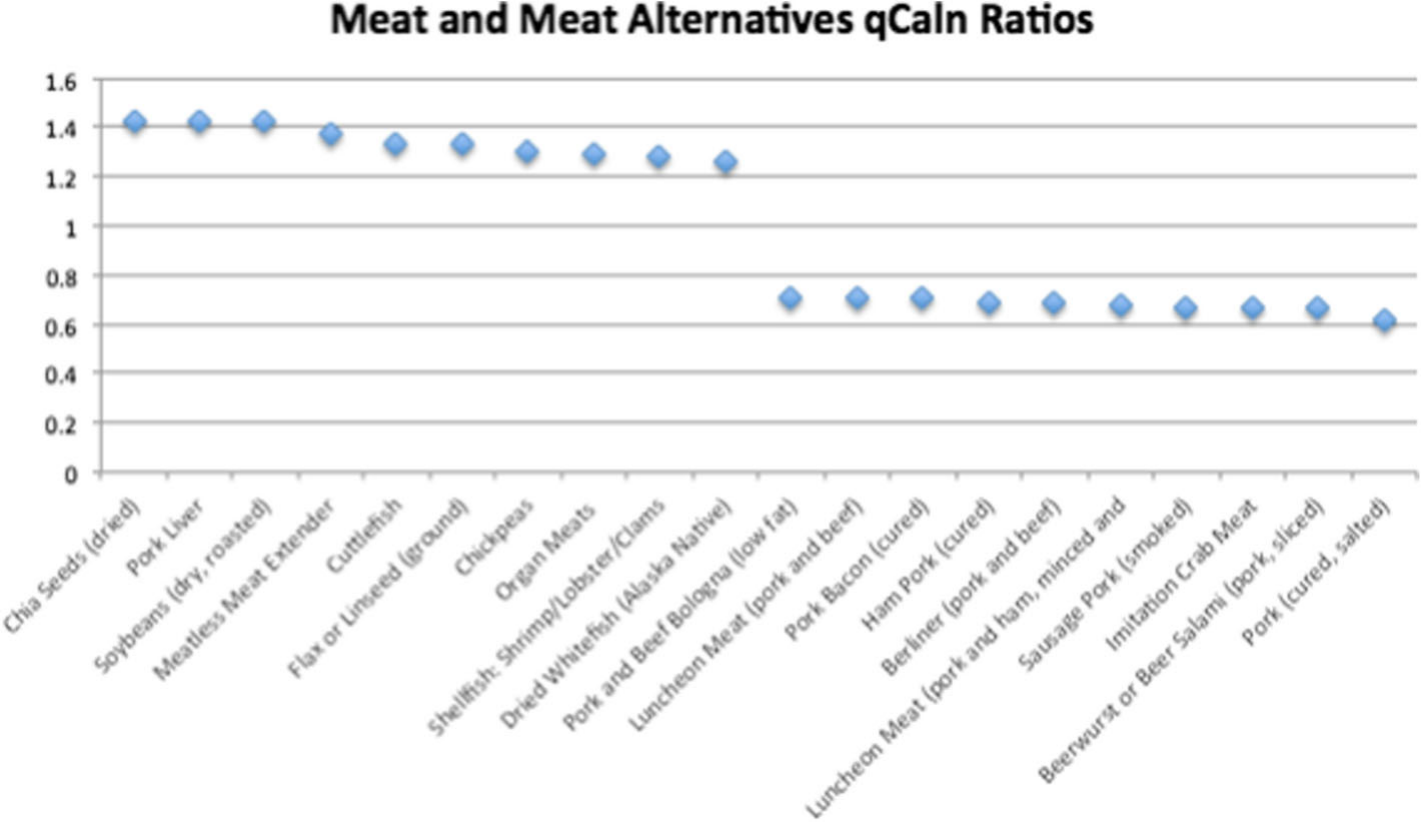
Spectrum of qCaln ratios for foods in the meat and meat alternatives food group

**Fig. 5:**
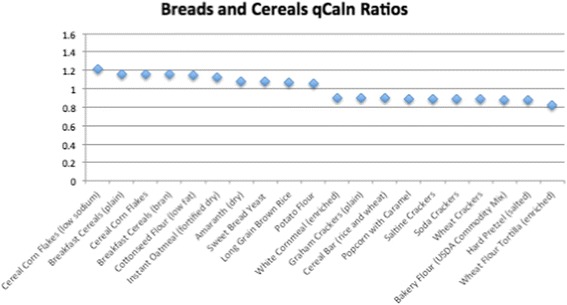
Spectrum of qCaln ratios for foods in the breads and cereals food group

Of the 1806 total food items analyzed, 534 had qCaln ratio values > 1.0 when compared to kilocalorie (Cal) content, and 1272 had ratios < 1.0. Ratios that are > 1.0 can be interpreted as higher quality food per calorie, whereas qCaln ratios < 1.0 represent lower micronutrient content per food item compared to its caloric content. Figures 1, 2, 3, 4 and 5 show the highest and lowest qCaln ratios (qCaln ratio = qCaln / kcal) for the vegetables, fruits and fruit juices, milk and dairy products, meats and meats alternatives, and breads and cereals food groups, respectively.

Figure 1 shows some of the highest 10 qCaln ratios and some of the lowest 10 qCaln ratios in the Vegetables food group (196 vegetables were analyzed for qCaln values). A table including the calculations of the qCaln ratio for each food item in the vegetables food group considered in this manuscript is included as Additional file 1.

Figure 2 shows some of the highest 10 qCaln ratios and some of the lowest 10 qCaln ratios in the Fruits and Juices food group (320 fruits and juices were analyzed for qCaln values).

Figure 3 shows some of the highest 10 qCaln ratios and some of the lowest 10 qCaln ratios in the Dairy food group (160 dairy products were analyzed for qCaln values).

Figure 4 shows some of the highest 10 qCaln ratios and some of the lowest 10 qCaln ratios in the meat and meat Alternatives food group (770 meat and meat alternative foods were analyzed for qCaln values).

Figure 5 shows some of the highest 10 qCaln ratios and some of the lowest 10 qCaln ratios in the Breads and Cereals food group (360 breads and cereals were analyzed for qCaln values).

In the case of vegetables, the highest 10 qCaln ratios included spinach, leeks, green peppers, collards, and turnip (greens), whereas those of the lowest qCaln ratios, reflecting lower micronutrient content to caloric content, were sweet pickles, pickled eggplants, canned capers, and red cabbage. For fruits, food items with the highest qCaln ratios were dried mixed fruits (prune, apricot, and pear), dry cranberry beans, wild raspberries, cherries, and blackberries, and those of the lower qCaln ratios were canned juices and fruits canned in syrup such as papaya, plums, and loquats. These results are in line with dietary recommendations that highlight the high nutrient yet low energy density of green leafy vegetables and other fresh fruits and vegetables that are not preserved in salt or sugar. It is worth noting that the sugar content of canned fruits and fruit juices was a major contributor to lower qCaln ratios for fruit items. When analyzing the dairy products, food items with the highest quality were low-fat, non-fat, and low sodium varieties of milk and cheese as these items score lower in terms of fat and thus caloric content and have lower sodium levels, which is found in abundance within dairy products and could contribute to elevated sodium intake levels.

**Fig. 6:**
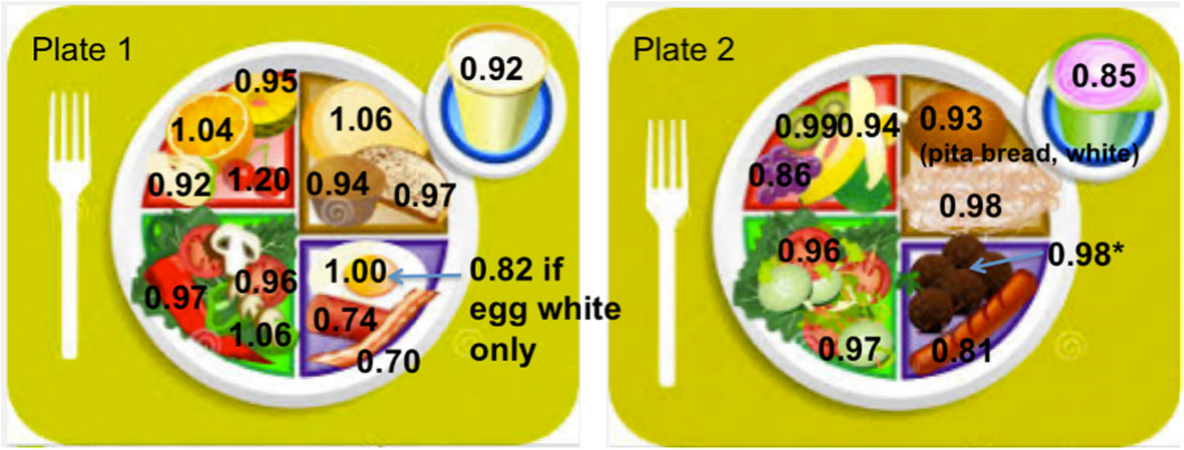
Comparison of two meals superimposed on the USDA MyPlate with qCaln ratios

Figure 6 displays a comparison of two meals superimposed on the USDA MyPlate along with each food item’s qCaln ratio. Where applicable, foods are assumed to be fresh. The bread in the right image is assumed to be white pita bread. The meatballs in the right plate are assumed to be meatless meatballs.

Finally, two separate meals were compared that would be considered good diet choices for each plate according to the MyPlate guidelines [8]. Note the generally higher values of qCaln ratios in Plate 1 compared to Plate 2. The average qCaln ratio for Plate 1 is 0.969, while the average qCaln ratio for Plate 2 is 0.927. Oranges and cherries have higher qCaln ratios (1.04 and 1.20, respectively) than grapes and bananas (0.86 and 0.94, respectively). For the meat and meat alternatives food group, eggs would be considered a better selection than meatless meatballs (1.00 to 0.98, respectively), but the hot dog on Plate 2 is a better choice than either the cooked sausage pork or bacon on Plate 1. However, as shown in the figure above, if the eggs on Plate 1 are made from egg whites only, then the qCaln ratio value drops from 1.00 to 0.82. Therefore, what is gained by reducing saturated fat (egg whites relative to eggs with yolks) is outweighed by the loss of various micronutrients contained in the egg yolk. The selection of whole milk in Plate 1 would also be a better selection than a yogurt cup (full fat variety) in Plate 2 with fruit flavoring (0.92 to 0.85, respectively).

## Discussion

This study aimed to develop a new unit of measure that accounts for both the caloric and micronutrient density of foods and to demonstrate the usability of this novel measure in assessing food quality. The qCaln was the standardized unit developed based on the caloric density and the amount and distribution of micronutrients per 100 g in a particular food. Results showed that food items in any food group can be analyzed based on their qCaln values. In addition, through calculating a qCaln ratio for each food item, the caloric and micronutrient contents of a food were compared with the food’s energy density (caloric value) in one single measure. Using the qCaln ratio, higher quality foods could be differentiated easily from lower quality foods. More specifically, the qCaln ratios of food items within each of the food groups, including vegetables, fruits/fruit juices, milk/dairy products, breads/cereals, and meats/meat alternatives, were compared to each other in this paper.

The advantages of the proposed approach over other existing food quality measures and nutrient profiling approaches lies in simplifying complex nutrient composition data into a singular based score that reflects both nutrient and energy density. The definition of nutrient dense foods have expanded over the years due to numerous attempts by researchers, regulatory agencies, and food companies to identify adequate criteria for ranking foods based on their nutrient composition. Some of the models highlighted the importance of limiting certain nutrients, such as total fat, saturated fat, sugar, or sodium, that were associated with adverse health outcomes, when defining nutrient-density [34, 35]. Other approaches emphasized recommended or desirable nutrients that may not be adequately consumed by population groups yet have favorable health benefits, including vitamins, minerals, and fiber [36], or included a combination of both, nutrients to be promoted and others that need to be limited [4, 6]. Despite the importance of these attempts in advancing the field of nutrient profiling, the majority of the approaches fell short of identifying a single measure to encompass the complexity of food and its nutritional value.

The selection of the 10 micronutrients that were included in the calculation of the qCaln accounted for two groups of nutrients: those that are not consumed in sufficient amounts and contribute, in part, to micronutrient deficiencies across various population groups [13–17] and less desirable nutrients that are consumed in abundance worldwide such as saturated fat, sugar, and sodium [18–21]. In addition, the number and choice of micronutrients considered in the qCaln calculation were similar, albeit not identical, to other nutrient profiling methods such as the NNR score, [26, 28], the NQI) [27] the RRRr food scores [22] and nutritional adequacy score of individual foods and limited nutrient score (SAIN/LIM system). Furthermore, the choice of the micronutrients in the present study was not dependent on nutrients that are consumed in low or high amounts in a specific country or context, but rather included a more comprehensive and global approach. This is in line with the recommendations from Drewnowski and Fulgoni, 2008 [4], who urged that the choice of nutrients included in nutrient profiling methods should be based on diets from different countries rather than focusing on a specific country or context, such as the United States. In fact, the formula for calculating qCaln presented in this study can be easily expanded or reduced based on objective research designed to identify the optimum number of micronutrients in nutrient profiling techniques.

It is worth noting that the percent daily value (%DV) of each micronutrient was not included in the calculation for qCaln within the present study for a number of reasons. First, Eqs. 3, 4, 5 and 6 described in the methodology ensure that the amount of a given micronutrient in a given food is compared to the normal distribution of that micronutrient relative to all foods within its food group. Therefore, dividing these contents by the %DV would lead to the same distribution and “S-score” (Eq. 6) used in the qCaln calculation. Second, the use of %DV relative to qCaln values of a particular food when compared to a food group would be better used for overall meal and diet construction, allowing for comparisons of foods between food groups. By accounting for the %DV in future calculations of the qCaln, this measure could be used for meal construction to educate consumers on making healthier dietary choices in an attempt to meet their daily dietary needs.

### Applications of the qCaln measure: nutrition education and dietary counseling

The proposed qCaln allows for a better interpretation of the food quality not only by researchers but also consumers. The qCaln can be linked to food-based dietary guidelines depicted visually through the MyPlate, MyPyramid, and the Mediterranean Diet Pyramid among other education models used by health care professionals to educate consumers about healthy dietary choices. For example, promoting the consumption of low-fat and low-sodium dairy products that have higher qCaln ratios (i.e. higher nutrient density with lower fat and sodium content as well as higher calcium and vitamin D), can assist consumers in meeting their beneficial nutrient requirements while avoiding less desirable nutrients. Similarly, promoting the consumption of fresh fruits like oranges rather than orange juice from frozen concentrate or sweetened fruit juices can help in making smarter food choices.

Training consumers to select foods with higher qCaln values, where appropriate, can help improve diet quality and meet dietary recommendations. Currently, dietary guidelines are presented to the public as recommendations in terms of serving sizes of food groups that are promoted to be consumed, such as whole grains, low fat dairy products, and foods to avoid including those high in sugar and fat. These recommendations are usually based on studies highlighting the daily optimal intakes of energy, and macro- as well as micro-nutrients among humans (acceptable macronutrient distribution ranges and DRIs, respectively). Despite the extensive efforts exerted by nutrition professionals to relay dietary guidelines in simple and interactive manners, consumers still face challenges in interpreting these recommendations. Difficulties arise when consumers are asked to make healthy dietary choices from a wide array of food products available on the market and with various nutrition facts labeling systems. In addition, deciphering the labeling systems, which include the amount of calories, macronutrient, and micronutrient content of foods, requires health and nutrition literacy at the consumer end, which may be lacking by various population groups and in numerous contexts. This is further complicated with the diversity of units, serving sizes, and nutrients listed on nutrition facts labels in differing countries, in addition to front-of-package labels along with other competing marketing slogans and techniques. Furthermore, fresh produce and certain types of meats and dairy products lack nutrition labels and the choice among the myriad of produce on the market leaves consumers confused as to which of the food items within each of the food groups are the most nutritious. The qCaln measure can merge the multiple nutritional measures including, caloric content of food, and the macro- and micronutrient content of food into one simple, standardized unit. In addition, the qCaln can be linked to optimal daily nutrient intakes of individuals and allows for daily meal construction. This linkage will allow consumers to make food purchases with conscious dietary knowledge rather than just preference, taste, convenience, and cost. The qCaln, if deemed valid in various contexts, could be used in addition to food labeling either on the front or the side of products, and on menus at various points of purchase with the potential for expanding nutrition education and supporting nutrition labeling systems that are already in place. Future research should evaluate the experiences of consumers towards the use of the qCaln and their success in attempting to make healthier dietary choices at different points of sales (supermarkets, restaurants, worksite cafeterias, schools, etc.).

Using the qCaln, as a composite score that takes into consideration energy and nutrient density, is not only valuable for consumers but also researchers and health professionals who seek to assess the diet quality of individuals and population groups. The proposed measure can provide a simple method to assess nutrient profiles of individual food items within the same food category while also providing investigators with a relatively simple tool to assess the overall nutrient profiles of individuals (for example, the average qCaln ratios of consumers over a specific period of time such as week or month). The latter measure can be then linked to other global measures of diet quality, such as the HEI and diet diversity scores.

### Applications of the qCaln measure: linking nutrition to agriculture

One of the main benefits and applications of the qCaln is to help individuals make better food item choices and improve their overall dietary intake. In fact, nutrition programs that attempt to increase awareness and proper consumption of micronutrients achieve some of the highest benefit-cost ratios, even in the short term [37]. However, improving dietary intake is not dependent only on the knowledge of the consumers regarding quality of foods through targeted nutrition awareness programs but also on the quantity and quality of agricultural produce available in the market and accessible to consumers. Nevertheless, the link between the evidence provided by these nutrition programs and agricultural practices is, at best, limited. In addition, there is a dearth in robust monitoring and evaluation tools to assess the impact of agriculture on nutrition outcomes [38]. Thus, it is important to generate easy-to-understand measures that can be compatible for both fields to best inform how to grow healthy food in a sustainable manner while respecting individuals and populations’ food choices, preferences, and traditions [39]. Researchers, public health professionals, farmers, and consumers can be trained to interpret the qCaln measure, as a relatively easier tool to use compared to other more complex agricultural and nutrition indicators. The qCaln could then be used to bridge the gap between nutrition and agriculture by tracking quality energy produced to quality energy consumed through the qCaln. However, the applicability of qCaln in linking agricultural management practices aimed at sustainable crop production with nutritional outcomes is worth more thorough explorations in future studies.

### Limitations

Findings from this paper need to be interpreted in light of a number of limitations. First, equal weights were assumed for all included micronutrients in the calculation for the qCaln. Although the use of equal-weighted scores has been used extensively by other researchers when developing nutrient profiling methods [4], targeted differential weightings for micronutrients have been suggested for context-specific cases such as biological quality of nutrients in food, bioavailability, and distribution of nutrients in the food supply [4]. Also, researchers have been showing the importance of altering the relative weightings of micronutrients in profiling to fit populations [40]. Thus, the formula for the qCaln can be further improved through reflecting different dietary needs and increasing or limiting intakes of specific nutrients for various segments of the population, such as increasing folate and vitamin B12 for pregnant women or limiting sodium and saturated fats for individuals at risk of hypertension or cardiovascular diseases.

Another limitation of the qCaln calculations in the present study is the lack of inclusion of bioavailability data and the effect of food preparation on micronutrient content of foods. For example, animal-based protein sources are more readily digested and absorbed allowing for higher bioavailability of essential micronutrients compared to plant-based sources, and cooked vegetables that are prepared through steaming have different micronutrient contents compared to boiled or baked vegetables [41]. To address these limitations, bioavailability and food preparation factors could be added to the proposed qCaln equation in future studies to allow for a better depiction of the quality of the foods and diets consumed by individuals. Additionally, the validity of this measure needs to be tested against other diet quality indices, including the HEI and diet diversity scores, and a number of nutrient profiling methods, such as the Nutrient-Rich Food Index (NRF) [6], and the SAIN/LIM scoring system [24]. The measure could also be tested against other objective health outcomes and biomarkers such as obesity, hypertension, and diabetes; and using more sophisticated validation techniques including ‘goodness of fit’ models (Drewnowski and Fulgoni 2008). Finally, further attention is needed to consider other attributes of food. For example, the qCaln could be compared to the Nutrient Rich Foods Index [7] to compare foods of varying food quality relative to cost. The qCaln, as a single number unit of measure, would allow for easy comparison of food quality to cost.

## Conclusion

The proposed unit of measure reflects a novel nutrient profiling approach that accounts for both the quantity of energy (caloric density) and the quality of nutrients in foods into one standardized metric and demonstrates the usability of this new measure to facilitate the assessment of food quality. Results show that the qCaln can be used to rank foods in terms of their nutrient and energy content simultaneously, making food selections within a food group easier to understand. This easy-to-use tool can be applied by public health professionals, regulatory agencies, and the food industry to promote the selection of healthier nutrient-dense yet low in energy content foods and assist consumers in meeting dietary guidelines and nutrition recommendations. In addition, this novel metric could be further validated by researchers in comparison to other nutrient profiling and diet quality measures to test its usability in assessing diet quality of individuals and population groups. This metric can have other applications and can be further expanded beyond the nutrition research serving as the impetus for nutrition-sensitive agriculture through tracking of food quality from production to consumption.

## Additional files

Additional file 1: Calculated qCaln ratios for each food item in the vegetables food group. (CSV 13 kb)

## Abbreviations

%DV: Percent daily value
AHEI: Alternative healthy eating index
DRIs: Dietary reference intakes
FBDG: Food-based dietary guidelines
HEI: Healthy eating index
NNR: Naturally nutrient rich score
qCaln: Quality of kilocalories for nutrition
SAIN/LIM: Nutritional adequacy scores of individual foods and limited nutrient score
USDA: United States department of agriculture
